# Alternative Splicing as a Target for Cancer Treatment

**DOI:** 10.3390/ijms19020545

**Published:** 2018-02-11

**Authors:** Nancy Martinez-Montiel, Nora Hilda Rosas-Murrieta, Maricruz Anaya Ruiz, Eduardo Monjaraz-Guzman, Rebeca Martinez-Contreras

**Affiliations:** 1 Centro de Investigaciones en Ciencias Microbiológicas, Instituto de Ciencias, Benemérita Universidad Autónoma de Puebla, Puebla 72470, Mexico; nancy.martinez.montiel@usherbrooke.ca; 2 Department of Microbiology and Infectious Diseases, Faculty of Medicine and Health Sciences, Université de Sherbrooke, Sherbrooke, QC J1E 4K8, Canada; 3 Facultad de Ciencias Químicas, Benemérita Universidad Autónoma de Puebla, Puebla 72470, Mexico; nora.rosas@correo.buap.mx; 4 Centro de Investigación Biomédica de Oriente (CIBIOR), Instituto Mexicano del Seguro Social (IMSS), Metepec, Puebla 74360, Mexico; manaya19@yahoo.com.mx; 5 Instituto de Fisiología, Benemérita Universidad Autónoma de Puebla, Puebla 72470, Mexico; eduardo.monjaraz@correo.buap.mx

**Keywords:** alternative splicing, cancer, diagnosis, therapeutics

## Abstract

Alternative splicing is a key mechanism determinant for gene expression in metazoan. During alternative splicing, non-coding sequences are removed to generate different mature messenger RNAs due to a combination of sequence elements and cellular factors that contribute to splicing regulation. A different combination of splicing sites, exonic or intronic sequences, mutually exclusive exons or retained introns could be selected during alternative splicing to generate different mature mRNAs that could in turn produce distinct protein products. Alternative splicing is the main source of protein diversity responsible for 90% of human gene expression, and it has recently become a hallmark for cancer with a full potential as a prognostic and therapeutic tool. Currently, more than 15,000 alternative splicing events have been associated to different aspects of cancer biology, including cell proliferation and invasion, apoptosis resistance and susceptibility to different chemotherapeutic drugs. Here, we present well established and newly discovered splicing events that occur in different cancer-related genes, their modification by several approaches and the current status of key tools developed to target alternative splicing with diagnostic and therapeutic purposes.

## 1. Introduction

mRNA processing is a key maturation process that includes mRNA splicing, polyadenylation and capping. Alternative splicing is a pivotal step in this maturation process that occurs in the nucleus in a co-transcriptional fashion and regulates eukaryotic gene expression. Although the extent of splicing was initially underestimated, it is currently established that almost 90% of the human genes undergo some splicing event, contributing to the enormous coding potential of the genome [1]. Consequently, defects in this mechanism can generate different diseases, including cancer [2]. 

Over the last two decades, molecular tools have been developed to correct or redirect alternative splicing events. Some of the strategies developed to modulate alternative splicing (AS) events include the use of short oligonucleotides or single stranded antisense oligonucleotides designed as a complementary molecule that targets a specific mRNA to regulate its expression both in vitro and in vivo [3]. In some cases, these oligos can recruit splicing regulatory factors, such as SR and hnRNP proteins [4]. More recently, microbial derivatives have shown the ability to modulate alternative splicing with effective anti-proliferative and anti-cancer activities [5]. Here, we summarize the current approaches developed to correct alternative splicing events that have shown an impact on cancer diagnosis and treatment. 

## 2. Alternative Splicing: The Mechanism

The general mechanism that regulates alternative splicing has been well established. In this process, exonic sequences are usually included, while the intronic portions are excluded from the mature messenger RNA and the particular combination of sequences retained or skipped produces a variety of combinations. The decision on which sequences are included or not relies on a combination of *cis* elements and trans-acting factors. The *cis* elements consist of general and regulatory elements that could be recognized by a large protein complex called spliceosome, comprised of more than 300 proteins and ribonucleoproteins [6]. The catalytic core of the spliceosome are the snRNPs (small nuclear ribonucleoproteins) U1, U2, U4, U5 and U6. These core factors interact with the general *cis* elements that include the 5′ and 3′ splice sites (ss), which correspond to the exon-intron boundaries at each side of the intron, a poly-pyrimidine tract that lies upstream of the 3′ ss and an adjacent element known as the branch point sequence (BPS). Alternative splicing decisions also involve the participation of auxiliary factors belonging to two major groups: the SR proteins [7] and the hnRNP family [8]. These proteins recognize additional regulatory elements coded in introns or exons. In each context, they could either inhibit or reinforce the recognition of a particular sequence, and, according to this function, they are called silencers or enhancers, respectively. In general, enhancers recruit SR proteins while silencers are usually recognized by hnRNP proteins [9,10]. However, the function of the *cis* and *trans* regulators of alternative splicing could change depending on the particular gene, the exon and intron sizes, the cellular context, the developmental state and the physiological requirements of the cell [11]. The final outcome of an alternative splicing event could be measured in terms of a global ratio, and, in several cases, this information could offer diagnostic information. Moreover, regulatory elements and factors could be the target for different molecules in order to modify particular splicing events with therapeutic purposes.

## 3. Molecular and Cellular Implications of Alternative Splicing Events in Cancer

It has been observed that a switch on particular alternative splicing events could occur in cancer related genes. This switch on alternative splicing could prevail during tumor progression and it usually correlates with an increase on cell proliferation and metastasis, which is the cause of 90% of all human cancer casualties [12]. It has been established that the alternative splicing events of different pre-mRNAs is altered during oncogenic progression and, in some cases, a relationship has been established between a particular splicing event and the development of some cancer features, like an increase in proliferation, vascularization and invasion [12,13], leading to the consideration of alternative splicing as a new hallmark of cancer [14]. In this scenario, the expression of a precise splicing isoform that is linked to tumor progression can be detected in normal tissues as well, but once that cell homeostasis is lost, alternative splicing provides a new source that contributes to tumor progression. 

Alternative splicing can be affected at different levels to produce changes that could correlate with an oncogenic state, including the altered activity, expression level or even mutations in regulatory splicing factors. This transition could occur due to changes in post-translational modifications, including phosphorylation [15,16], methylation [17,18] and sumoylation [19] of different splicing factors, with the concomitant impact not only on splicing regulation, but also on different aspects of cell biology. Moreover, somatic mutations in genes coding for components of the splicing machinery could also contribute to the development of tumors. According to the information deposited in the databases of the International Cancer Genome Consortium (ICGC), it seems that approximately 300 splicing-related genes are mutated in all types of cancer, where the most frequently mutated genes include several hnRNP (NOVA1, hnRNP M, hnRNP C, hnRNP A2/B1, hnRNP F, and RALY) and SR proteins (SRSF4, RBM39, Tra2α, and Tra2β) together with SR-protein kinases (SRPK1 and SRPK2) and RBM proteins (RBM4 and RBM5). Interestingly, the snRNPs which are the core components of the spliceosome seem to be rarely affected in cancer, while several mutations could be found in some cancer patients only at the *SF3B1*, *U2AF1* or *SNRNP70* genes [20,21,22,23,24,25]. All these observations strongly support the relevant participation of alternative splicing in cancer, but the precise mechanism that governs the role of each splicing factor in different types of cancer remains to be elucidated.

Concerning the expression levels for different splicing factors in the context of cancer, it has been demonstrated that several splicing factors are overexpressed in different human cancers [26], such as *SRSF1* and *SRSF3* that show high expression levels in several human cancer types, while the silencing of these genes can lead to apoptosis in various cancer cell lines [27,28]. hnRNP A1 and hnRNP A2 have been long related to cancer regulation given their ability to recognize and protect telomeric sequences [29]. These factors are also overexpressed in a wide variety of cancers and the silencing of these genes induces apoptosis in cancer cells but not in normal cells [30,31,32]. hnRNP A1 or A2 can regulate more than 2000 alternative splicing events [33] and some of these events are related to cellular abnormalities relevant for tumor progression [34]. High levels of hnRNP I have been found in gliomas [30,35] and this splicing factor may be involved in the progression of astrocytic tumors [36]. hnRNP H is also overexpressed in gliomas and the silencing of this gene produces apoptosis in U373 (glioma) and HeLa cells [37]. Along the pre-mRNA, binding sites for hnRNP F shape secondary structures named G-quadraplexes, which regulate the AS of CD44 resulting in the regulation of epithelial–mesenchymal transition (EMT). G-quadraplexes seem highly prevalent in breast cancer patients and correlate with patient survival [38]. Some molecules that bind to and stabilize G-quadraplexes have shown to induce cell death, preferentially in cancer cells [39]. 

On the other hand, several splicing factors have been related to an oncogenic phenotype, including SRSF1 [40], SRSF9 [41], hnRNP A1/A2 [32] and hnRNP H [42]. For example, overexpression of SRSF1 have shown the ability to induce the formation of sarcomas in nude mice [40]. Moreover, key regulators of cell cycle progression like Cyclin D1 and H-ras have shown key alternative splicing profiles with different oncogenic activity. Cyclin D1 regulates cell cycle progression through its association with CDK4/6 [43]. The more abundant pre-mRNA is the full-length product Cyclin D1a, where the 5 exons are included. Cyclin D1b is generated due to the recognition of a polyadenylation site in intron 4 [44] and this isoform is overexpressed in breast and prostate cancer [45,46]. Each isoform displays different cell localization: while Cyclin D1b resides in the nucleus, D1a shuttles between the nucleus and the cytoplasm according to cell cycle progression and it has been shown that nuclear localization correlates with a more oncogenic activity [47]. Regarding the *cis* elements and *trans* acting factors that regulate AS of Cyclin D1b, a polymorphism (G870A) located near the 5′ ss of exon 4 may affect the recognition of this exon, favoring production of isoform D1b [44,48]. In addition, functional binding sites for Sam68 and SRSF1 have been identified [49,50]. Cyclin D is one of the several examples of cancer-related genes showing differential splicing profiles in tumor vs. normal tissue; additional examples of alternative splicing events that display a similar behavior are shown in Table 1. Finally, full cellular networks seem to be influenced by changes in AS regulation in the context of cancer cells, as has been depicted for the DMP1-ARF-MDM2-p53 pathway [51] and during the epithelial–mesenchymal transition [52].

All this information supports the notion that alternative splicing events are determinant for cancer progression, with direct implications in every aspect of cell biology, including the following: modulation of gene expression, chromatin reorganization, cell cycle control, cellular metabolism, regulation of intracellular signaling cascades and apoptosis [53,54].

In the last few years, there has been an extensive effort to determine which mutations could be responsible for changes in alternative splicing events that correlate with a particular type or stage of cancer [72,73,74]. Unfortunately, in some cases it has been difficult to establish a precise correlation between one mutation and the correspondent alternative splicing event due to the transitive nature of gene expression. Some specific mutations that lie in splicing sites or regulatory elements have been annotated in different databases like the Tumor Portal for various cancer-related genes. Interestingly, these mutations have been discovered in samples recovered from patients with different types of cancer (Table 2). However, further insights are required to uncover the functional implications of these mutations and the role of particular splicing isoforms in tumor progression and in different types of cancer. 

## 4. Small Molecules That Modulate Splicing with Potential in Cancer Treatment 

The relevance of RNA splicing in cancer is rapidly emerging. Small molecule inhibitors acting at different levels of the splicing process were initially discovered as chemical probes to study splicing regulation in vivo or in vitro. However, some of them resulted useful for the treatment of several human diseases such as cancer. 

FR901464 is a natural product considered the prototype compound for splicing inhibitors with an antitumor activity [75,76]. In HeLa cells, FR901464 inhibits pre-mRNA splicing with an IC_50_ of 0.05 μM acting on splicing factor 3b (SF3b) [77,78]. FR901464 has a potent anti-proliferative effect against multiple human cancer cell lines such as breast cancer MCF7, lung adenocarcinoma A549, colon cancer HCT116, colon cancer SW480 and also against the murine leukemia P388 with IC_50_ values of 1.8, 1.3, 0.61, 1.0, and 3.3 nM, respectively. Moreover, FR901464 exhibited a prominent effect at doses of 0.056–1 mg/kg against human solid tumors implanted in mice while inhibiting tumor growth in various xenograft models and it has been shown that this splicing inhibitor promoted G1 and G2/M phase arrest in the cell cycle by the splicing inhibition of p27 [77] and suppressed the transcription of some inducible endogenous genes as c-Myc. FR901464 has been modified to increase its anti-proliferative or anti-tumor activities and among the structural analogs designed, 1-Desoxy FR901464 retains its anti-proliferative activity being more active against Jurkat cells than the original molecule [79]. 

Spliceostatin A (SSA) is a derivative of FR901464 that exhibits important anti-proliferative and anti-tumor activities [80] affecting the splicing patterns of cell cycle regulators such as Cyclin A2 and Aurora A kinase [81], inducing the accumulation of cells in the G_2_/M phases of the cell cycle [75,82]. In HeLa cells, SSA inhibits splicing with an IC_50_ of 0.01 μM [83]. Several studies have shown that SSA A inhibits both in vivo and in vitro splicing and promotes pre-mRNA accumulation by a nonproductive recruitment of U2 snRNP of subunit SF3b [84]. Therefore, SSA inhibits spliceosome assembly by slowing the A to B complex transition. This inhibition requires functional *cis* elements, including the 5′ splice site and branch point adenosine in the pre-mRNA with decoy sequences upstream of their productive binding site at the branch point sequence, and also the *trans* acting factors U1 and U2 snRNPs in the presence of ATP enabling the interference with the spliceosome subsequent to U2 snRNP addition [77,85,86]. Supporting this mechanism, in a fission yeast strain deficient of the multidrug resistance protein Pmd1, SSA also inhibits splicing and nuclear retention of pre-mRNA by SF3b complex [83]. 

A potent analog of FR901474 is Meayamycin. When tested in breast cancer MCF-7 cells, the GI_50_ values determined were 10 pM for the analog Meayamycin and 1.1 nM for FR901464, showing that the analog was 100 times more potent than the molecule depicted originally in this cellular context [87]. The analysis of *MCL1* splicing using the combination of Meayamycin B with the bcl-xL inhibitor ABT-737 showed an efficient modulation of alternative splicing in A549 and H1299 cells. At the same time, this combinatorial treatment induced apoptosis in both cell lines [88,89]. Meayamycin also inhibited pre-mRNA splicing in the HEK-293 cell line [90]. The target of Meayamycin B is SF3b1 and it acts as a splicing inhibitor by impairing the transition from the complex H to the complex A [88]. 

Isoginkgetin (7-*O*-β-d-glucopyranoside), isolated from dried leaves of *Gingko biloba*, is a glycosylated biflavonoid that inhibits splicing both in vivo or in vitro. The exposure of U2OS and HeLa cells to Isoginkgetin for 2–16 h lowered their splicing capacity by as much as 75% and it was able to decrease tumor invasion [91,92]. The mechanism described for the splicing inhibition by Isoginkgetin consists in the stable recruitment of the U4/U5/U6 tri-small nuclear ribonucleoprotein, which was shown using in vitro splicing reactions and HeLa cells, inhibiting the transition from spliceosomal complex A to B at a concentration of 30 nM with the concomitant accumulation of the pre-spliceosomal complex A [93]. Isoginkgetin treatment produces a nearly two-fold increase in the time required for lariat formation and intron processing time, and it blocks the formation of spliceosomal complex B [94]. 

Pladienolide B (PB) is a macrocyclic lactone with anti-proliferative activity [95] on several cell lines such as BSY-1, PC-3, OVCAR-3, DU-145, WiDr, DLD1 and HCT-116 [96]. PB induces cell cycle arrest at both G1 and G2/M and inhibition of mouse xenograft [97], and it has anti-tumoral activity on gastric cancer cell lines and primary cultured cancer cells from carcinomatous ascites of gastric cancer patients. The mean IC_50_ value of pladienolide B was 4.9 ± 4.7 nM. In addition, it has been related to an increase on the expression of p16 and cyclin E genes while inducing apoptosis [98]. PB affects both in vitro and in vivo splicing at the level of the spliceosome SF3b1 subunit. Exposure of cells to PB for 2–16 h lowered their splicing capacity up to 75% [99].

FD-895 is a PB analog macrolide antibiotic isolated from *Streptomyces hygroscopicus* strain A-9561 [99] with cytotoxic activity against several types of cancer cells such as Adriamycin-resistant HL-60. In patients with chronic lymphocytic leukemia, FD-895 induces intron retention and promotes apoptosis. This analog also inhibits splicing by interacting with the SF3b subunit [100]. 

Herboxidiene also known as GEX1A is a polyketide recovered from *Streptomyces chromofuscus* A7841 with the ability to induce both G1 and G2/M cell cycle arrest in human normal fibroblast cell line WI-38, A549, JeKo-1 and WiDr cancer cell lines [101]. It has been shown that Herboxidiene modulates alternate splicing of the MDM-2 pre-mRNA [102]. GEX1A inhibits constitutive and alternative splicing by targeting SF3b1, blocking the association of SAP155 in SF3b [103] and disrupting the transition from A to B complex in the spliceosome assembly pathway [104].

Sudemycin E is a synthetic analog of FR901464 [100] that affects the alternative pre-messenger RNA splicing in a global manner and stops the growth of tumors in mice by targeting mainly cancer cells. Some of the pre-mRNAs affected by this molecule are *RPp30*, *DUSP11*, *SRRM1*, *PAPOLG*, *MLH3* and *IBTK* genes [105]. The inhibitory effect on splicing occurs by its association to the U2 component SF3b1 which fails to maintain a tri-methylated state in actively transcribed genes [106]. In addition, Sudemycin E promotes global changes in gene expression and the arrest in the G2 phase of the cell cycle. This drug induces a selective cytotoxicity in primary chronic lymphocytic leukemia (CLL) cells in combination with ibrutinib. In a different study, after 48 h of treatment, Sudemycin E showed an anti-proliferative effect on HeLa, HEK293 and Rh18 cells with a IC_50_ of 0.16, 12.85 and 1.12 μm, respectively [106]. 

Sudemycin D6 is a stable derivative of Sudemycin E but with no splicing inhibition activity. However, it can alter alternative splice site selection at low μM concentrations in HeLa, RH19, and HEK293 cells [106]. Sudemycin D6 also binds to the splicing component SF3B1. In the Rh18 cell line and in the JeKo1 mantle cell lymphoma tumors, Sudemycin D6 showed a potent modulation of the alternative splicing of MDM2. Similar to Sudemycin E, this molecule exhibits cytotoxic activity on SK-MEL-2, JeKo-1, HeLa, SK-N-AS, and PC-3 cells with IC_50_ of 39, 22, 50, 81 and 142 nM, respectively [107]. Recently, *DUSP11* and *SRRM1* genes were identified as biomarkers for Sudemycin D6 treatment in human blood [108]. Another stable and more potent derivative of Sudemycin E is Sudemycin K, which produces MCL1-exon2 skipping in HeLa cells. The cytotoxicity of Sudemycin K has an IC_50_ 2.3 ± 0.81 compared to Syd E of 764 ± 113 [109]. 

Additional splicing inhibitors with potential as anticancer drugs are Thailanstatin A (TST-A), Thailanstatin B (TST-B), Thailanstatin C (TST-C) and Thailanstatin D (TST-D), which were isolated from the fermentation broth of *Burkholderia thailandensis* MSMB43. TSTs inhibited in vitro splicing with half-maximal inhibitory concentrations even in the sub-μM range. Thailastatins (TSTs) associate to the SF3b subcomplex in the U2 snRNP particle of the spliceosome, preventing base-pairing interactions with sequences located at the 5′ of the branch point [110]. TSTs display anti-proliferative activities in cancer cell lines. TST-A has an IC_50_ of 1.11, 2.26, 2.58, and 2.69 μM on DU-145, NCI-H232A, MDA-MB-231 and SKOV-3 cells, respectively. On the other hand, TST-D showed an IC_50_ of 6.35, 7.56, 9.93, and 7.43 μM on DU-145, NCI-H232A, MDA-MB-231 and SKOV-3 cells, respectively [111]. 

4bHWE is a molecule isolated from *Physalis peruviana* with a potential role as anticancer drug and it has shown a global impact on alternative splicing by a decrease on the phosphorylated form of the splicing factor SRSF1 with a concomitant increase of the levels of H3K36me3 while modifying chromatin condensation [112]. Interestingly, this inhibitor affects several apoptotic genes as *HIPK3*, *SMAC/DIABLO*, *SURVIVIN*, *AIMP2*, *BCL2L11*, *BIRC5*, *CASP3*, *CEACAM1*, *CPE*, *FGFR2*, *FN1*, *FPGS*, *HIF1A*, *KLF6*, *MCL1*, *MDM2*, *MKNK2*, *TERT*, and *VEGFA* [41]. 4bHWE alters the cell cycle by promoting G2/M arrest and the induction of apoptotic cell death with a treatment of 5 μg/mL for 24 h. Albeit, it can induce the sub-G1 accumulation in a dose-dependent manner. 4bHEW has an anti-proliferative effect on the human lung cancer cell line H1299 with an IC_50_ of 0.6 and 0.71 μg/mL, for 24 and 48 h, respectively [113]. 

In different types of cancer, the expression of CK2 is abnormally elevated. CX-4945 or Silmitasertib is an inhibitor of casein kinase 2 (CK2) and a molecule currently in clinical trial (Phase II) for cancer treatment, specifically on solid tumors and hematological malignancies. Silmitasertib exerts anti-proliferative effects in hematological diseases by decreasing CK2 expression and suppressing activation of CK2-mediated PI3K/Akt/mTOR signaling pathways [114]. Such effects on cell proliferation were induced by an abnormal alternative splicing of CK2 pre-mRNA. CX-4945 modulates the phosphorylation state of SR proteins by targeting Cdc2-like kinases (Clks) in an ATP-competitive manner. This drug has an IC_50_ on Clks of 3–90 nM [115]. CX-4945 impairs the growth of *Candida albicans* and for this reason it can also control candidiasis, which appears to be associated with cancer treatment [116]. 

Borrelidin (BN) is a natural polyketide that inhibits bacterial and eukaryal threonyl-tRNA synthetase with multiple applications such as antibacterial, antifungal, antimalarial, insecticidal, herbicidal and anticancer activities, being a potent inhibition of angiogenesis and metastasis [117,118]. BN exhibits an anti-proliferative effect on the malignant acute lymphoblastic leukemia Jurkat and CEM cell lines, where it induces apoptosis [118]. In colon tumor cells, the spliceosome-associated protein FBP21 (formin binding protein 21) was the target of borrelidin. BN alters the ratio of vascular endothelial growth factor (VEGF) isoforms in retinal pigmented endothelial (RPE) cells in favor of anti-angiogenic isoforms [119]. 

Recently, the ability to inhibit splicing and an anti-cancer activity have been associated with the following drugs: clotrimazole, flunarizine, and chlorhexidine. Chlorhexidine is a selective inhibitor of specific Clks that phosphorylate SR proteins and it affects the alternative splicing of many pre-mRNAs including RON, caspase 9, and HIV Tat2-3 [120]. Finally, an interesting compound with potential anti-cancer activity is DDD00107587 or Madrasin, a small molecule that inhibits the formation of the spliceosome in subsequent steps to complex A formation. In HeLa and HEK293 cells, Madrasin produces a weak exon skipping on AURKA, CCNA2, and p27 mRNAs. After 24 h treatment with 30 μM of DDD00107587, there is a cell cycle arrest with 30% of cells in S phase and 24% in G2 and M phases [121]. All this evidence indicates that existing and novel small molecules that modulate splicing will be a real option for cancer treatment with important repercussions in the health system. 

Currently, the majority of the experimental approaches devoted to analyzing the effect of different small molecules on alternative splicing regulation involve the use of human cancer cell lines. However, new systems could prove useful for these kind of studies, such as primary cell cultures obtained from cancer patients. In this regard, only few studies evaluate the effect of splicing modulators using primary cultures, but the observations so far confirm that the effects induced by splicing modulators on cell proliferation and on splicing regulation could be observed also in this cell context. For example, it was shown in a recent study that pladienolide B has very high antitumor activity not only against cultured cell lines, but also against primary cultured cells from patients with gastric cancer [98]. Additional studies involving the use of primary cultures could provide further insights into the effect of small molecules in the cellular context of cancer patients.

## 5. Antisense Oligonucleotide Technology Applied to Modulate AS Events

Antisense oligonucleotides (ASOs) are short oligonucleotides usually of 15–25 bases that correspond to the sequence complementary to a specific RNA transcript. To modify an alternative splicing event, an ASO could be directed to regions located at or close to a splice site, masking normal or aberrant splicing events leading either to exon exclusion or inclusion. Due to their high specificity, ASOs are a versatile tool that can be used to modify RNA expression with therapeutic purposes. In cancer treatment, antisense oligonucleotides can be used to shift a splicing event towards an anti-proliferative isoform in a very specific and directed manner. In a different approach, alternative or aberrant splicing can be modified by the steric blocking of antisense molecules, known as splice switching oligonucleotides (SSO), which results in restoring the production of favorable splicing variants, with potential benefits in cancer treatment [122,123,124,125,126,127,128,129,130]. SSOs consist of short oligonucleotides directed to target specific *cis* elements within the pre-mRNA, and they compete with splicing factors to access these elements, contributing to exon and intron definition. SSO technology has been applied to modulate AS events for different cancer-related genes. For example, SSOs were designed to the proximal 5′ ss of bcl-x pre-mRNA to block splicing at this site and induce a splicing switch favoring the production of the short apoptotic isoform. A decrease in bcl-xL mRNA and protein, accompanied by an increase in bcl-xS mRNA and protein was observed various cancer cell lines treated with these SSOs in a dose-dependent and sequence specific manner [131]. Bcl-x SSO also showed the ability to induce apoptosis, mainly in PC3 cells [132], while sensitizing MCF7 and A159 cells to apoptosis associated to chemotherapeutic agents or UV radiation [132,133,134]. The first demonstration of SSO efficacy in tumors in vivo corresponds to the SSO-induced shift from bcl-xL to bcl-xS in a mouse model of melanoma lung metastases where the tumor burden was reduced [135]. Effective splicing regulation mediated by SSO technology has also been accomplished for HER2 [63], FGFR1 [136], ATM [137] and PSMA [138] pre-mRNAs. 

Particular applications of SSO technology are the ESSENCE (Exon-specific splicing enhancement by small chimeric effectors) and TOSS (Targeted oligonucleotide silencers of splicing) molecules. The ESSENCE is an approach that uses an oligoucleotide bound to a peptidic RS-domain at the 3′ end designed to enhance exon inclusion mediated by SR proteins [139]. This method corrected AS of BRCA1 exon 18 [140] and bcl-x [141]. On the other hand, TOSS strategy employs an oligonucleotide with a 5′ or 3′ extension that is recognized by hnRNP A1/A2 proteins, which strongly inhibits splicing at the targeted splice site; this approach was also effective in bcl-x alternative splicing modulation [142].

Classical antisense oligonucleotides (ASO) form double-stranded hybrids that serve as targets for RNase H [143,144] and it consists on a molecule of 17–25 nucleotides length [145] with a specific target sequence of around 20 nucleotides that confers high specificity [146]. However, modulatory oligonucleotides like ASOs or SSOs have been modified mainly to increase their resistance to degradation. Some of these modifications include peptide-nucleic acids (PNAs), phosphorodiamidate morpholino oligos (PMOs) and 2′-OMe (2′-orhto-methyl) extensions. 

Delivery methods for oligonucleotide technology are also necessary to introduce the nucleic acid or analogous molecule into the cell. Some molecules that have been effectively used as carriers are the cell-penetrating peptides (CPPs), which are modified molecules based on antimicrobial peptides. CPPs are a group of efficient non-viral delivery vectors that mediate the entry of a variety of molecules used in gene modulation, both in vivo and in vitro and several splicing-regulatory oligonucleotides have been conjugated to CPPs like Penetratin, and Transportan with high efficiency [147].

In the past few years, several antisense drugs have been approved by the U.S. Food and Drug Administration (FDA), including Fomivirsen for the treatment of cytomegalovirus retinitis [148], Mipomersen for homozygous familial hypercholesterolemia [149], Eteplirsen for Duchenne muscular dystrophy [150], and Nusinersen for spinal muscular atrophy [151]. This last example is actually an alternative splicing regulator and is probably the strongest evidence supporting the possibility to use alternative splicing as a target to treat different disorders. Several studies have demonstrated that phosphorothioate and 2′-*O*-methoxyethyl- modified ASOs targeting the splicing *cis* element called ISS-N1 increase SMN2 exon 7 inclusion, thus increasing levels of SMN protein, alleviating the symptoms in patients with spinal muscular atrophy [152]. This treatment has demonstrated significant extensions in life expectancy and lead to the approval of the treatment in USA and Europe. Under this scenario, the possibility of antisense oligonucleotide technology to become a reality for cancer treatment by targeting alternative splicing seems plausible in the upcoming years.

## 6. Recent Approaches Targeting Alternative Splicing for Cancer Treatment

The evidence generated from the study of alternative splicing events and their role in different types of cancer have originated the development of different tools with therapeutic purposes. Some of these efforts have been conducted to the proposal of different patents that have been registered and could hopefully be applied in future biotechnological practices (Table 3). 

All these efforts indicate that the application of the findings related to alternative splicing regulation could be used with therapeutic purposes for different types of cancer in the clinic. Another piece of evidence suggesting that alternative splicing could be a reliable target for cancer treatment in the near future arises from the fact that different studies that involve the use of antisense therapy in combination with other anti-cancer therapies are being tested in clinical trials at different levels (Table 4). With this evidence, we could anticipate that, hopefully in the near future, effective modulators of RNA splicing would be developed and the approaches towards targeting alternative splicing as a real therapeutic strategy for cancer treatment are already on the way. 

## 7. Conclusions

Alternative splicing has rapidly emerged as an attractive target for pharmaceutical developments. An extensive amount of information arising from genome-wide studies have uncovered the general implications of alternative splicing events in several aspects of cell physiology, particularly in the different pathways involved in cancer biology. However, future efforts are still needed to uncover the existing connections between the regulation of gene expression and the cellular behavior generated. To this end, multidisciplinary approaches may help to gain further observations from the already existing information. Interestingly, recent advances are close to reaching the clinic and the general public, suggesting that alternative splicing-modifying tools could be useful under regular basis for cancer diagnosis and therapeutic in the upcoming years.

## Figures and Tables

**Table 1:** Alternative splicing isoforms related to tumor progression.

Gene	Description	AS Event	Role in Cancer	References
*BIN1*	This gene encodes several isoforms of a nucleocytoplasmic adaptor protein, one of which was initially identified as a Myc-interacting protein with features of a tumor suppressor.	Alternative exclusion/inclusion of the cassette exon 7. This shorter variant is also called IId and S1/R3-6 and binds dynamin, synaptojanin, and clathrin.	Caspase-independent apoptotic activation is impaired when aberrant isoforms are expressed.	[55,56 ,57]
*CASP8*	Member of the caspase family, which may interact with Fas-interacting protein FADD.	Alternative exclusion/inclusion of the cassette exon 4.	This protein is involved in apoptosis induced by Fas and various apoptotic stimuli.	[58,59]
*ENAH*	Response factor to mitogenic stimuli, such as EGF that triggers MAPK activation.	Alternative exclusion/inclusion of the cassette exon 12.	Functional role of hMena + 11a in breast cancer cell proliferation.	[60,61]
*ERBB2*	Member of the epidermal growth factor (EGF) receptor family of receptor tyrosine kinases that lacks the binding domain for growth factors. However, it can heterodimerize with other ligand-bound EGF receptor family members, enhancing kinase-mediated activation of downstream signaling pathways, such as those involving MAPK and IP3K.	Alternative start site, where isoform a corresponds to the full length, while isoform b shows a shorter N-terminus. Alternative exclusion/inclusion of the cassette exon 5.	Amplification and/or overexpression of this gene has been reported in numerous cancers, including breast and ovarian tumors.	[62,63, 64,65,66]
*FGFR1*	Member of a family of trans-membranous receptors that possess an extracellular domain composed of three Ig-like domains, a single transmembrane helix-domain and an intracellular domain with tyrosine kinase activity.	Alternative site at the 5′ UTR, that generates the use of an alternative promoter, the inclusion of an alternate exon, the use of an alternate translation start site, and uses an alternate in-frame splice site, lacking two internal segments.	Key roles in proliferation, differentiation, and tumorigenesis.	[67]
*PLAUR*	uPAR was originally identified on the monocyte-like human cell line U937 as the membrane receptor for the serine protease urokinase-type plasminogen activator (uPA).	Alternative exclusion/inclusion of the cassette exon 5, which partially covers domain II of the receptor. The short splice variant lacking exon 5 has prognostic relevance in breast cancer.	Implicated in cancer invasion and metastasis.	[68]
S100A4	The protein encoded by this gene is a member of the S100 family of proteins containing 2 EF-hand calcium-binding motifs.	Variant 1 has an alternate 5′ UTR, compared to variant 2. Both variants 1 and 2 encode the same isoform.	Involved in the regulation of several cellular processes such as cell cycle progression and differentiation.	[69]
*SYK*	Member of the family of non-receptor type Tyr protein kinases. Widely expressed in hematopoietic cells and involved in coupling activated immunoreceptors to downstream signaling events that mediate diverse cellular responses.	The short isoform lacks the alternative in-frame exon 7, resulting in isoform Syk (S). The longest isoform Syk (L) corresponds to the full-length transcript.	Involved in proliferation, differentiation, and phagocytosis, it is considered a modulator of epithelial cell growth and a potential tumor suppressor in human breast carcinomas.	[70,71]

**Table 2:** Alternative splicing-associated mutations in cancer-related genes.

Gene	Chr	Cancer Type *	No. Mut	Position	Ref Allele	New Base
*APC*	5	CRC	10	112128143, 112116601, 112173251, 112128143, 112128143, 112128143, 112128143, 112136975, 112128143, 112128143	C, G, C, C, C, C, C, G, C, C	T, A, T, T, T, T, T, A, T, T
		LUAD	3	112170646, 112170864, 112111325	A, T, G	G, C, T
*ARID1A*	1	BRCA	1	27099478	G	A
		ESO	1	27100207	C	T
		LUAD	1	27102066	A	G
		LUSC	3	27100389, 27092947, 27092948	G, G, G	A, C, T
		UCEC	6	27100207, 27088642, 27057642, 27100207, 27100207, 27100207	C, G, G, C, C, C	T, T, T, T, T, T
*ATM*	11	BLCA	1	108196036	G	A
		BRCA	4	108151895, 108202170, 108160511, 108186737	G, G, TCACTATATCAACCAAAGGTAAATAACA, G	A, A, -, A
		CLL	2	108172374, 108200943	G. A	T, C
		CRC	1	108218092	G	C
		DLBCL	1	108190784	A	G
		ESO	1	108202170	G	A
		HNSC	3	108141873, 108121428, 108236053	C, G, G	T, C, A
		KIRC	2	108141874, 108206572	G, G	A, C
		LUAD	4	108199966, 108175579, 108205695, 108119829	G, G, G, G	A, T, T, T
		LUSC	1	108098502	G	A
		MEL	1	108106398	A	G
		UCEC	4	108186548, 108198485, 108158326, 108175401	A, G, G, G	G, T, T, A
*BIN1*	2	LUAD	1	127811020	G	C
		BLCA	1	127834283	C	T
*BRAF*	7	LUAD	4	140453193, 140453193, 140508693, 140494268	T, T, C, C	C, C, A, A
		LUSC	1	140449220	T	C
		MM	1	140453193	T	C
		OV	1	140453193	T	C
*CASP8*	2	HNSC	1	202151181	G	C
		LUAD	1	202137501	T	G
		UCEC	4	202137666, 202150039, 202137500, 202137360	G, C, G, G	A, T, A, T
		HNSC	3	202137620, 202137359, 202150039	G, A, C	C, G, T
*CDKN2A*	9	BLCA	1	21971209	T	A
		CRC	1	21970974	C	T
		HNSC	9	21971208, 21971209, 21968242, 21968242, 21971208, 21968243, 21968242, 21970900, 21970900	C, T, C, C, C, T, C, CC, C	T, A, T, T, T, C, T, TT, A
		KIRC	1	21970901	C	T
		LUAD	3	21968242, 21971208, 21971209	C, C, T	A, A, A
		LUSC	4	21968243, 21994137, 21994138, 21970901	T, CC, C, C	A, GA, A, T
*CREBBP*	16	BRCA	2	3790551, 3827658	C, T	A, C
		DLBCL	3	3808853, 3817719, 3807288	A, A, C	G, C, A
		HNSC	2	3786204, 3820572	C, G	A, C
		KIRC	1	3843626	G	A
		OV	1	3789724	A	C
*EGFR*	7	GBM	1	55268881	G	A
		LUAD	2	55231427, 55220240	G, G	C, T
		LUSC	1	55220237	A	G
		MM	1	55221845	C	A
		NB	1	55240678	C	A
*ENAH*	1	LUAD	1	225702299	C	T
		CRC	1	225692754	G	A
		UCEC	1	225695652	C	T
*ERBB2*	17	ESO	2	37880261, 37880261	G, G	T, T
		MEL	1	37872858	G	A
		LUAD	1	37876038	A	T
		LUSC	1	37866733	C	T
		KIRC	1	37876087	G	C
*FAT1*	4	HNSC	4	187531171, 187535344, 187524190, 187534263	T, C, T, C	A, T, C, T
		KIRC	1	187521052	C	T
		LUAD	2	187510374, 187527368	C, C	A, G
		LUSC	1	187535499	C	G
		UCEC	3	187521515, 187530956, 187530956	C, G, G	G, A, A
*FBXW7*	4	CRC	3	153249361, 153258955, 153332456	T, T, T	1-, A, G
		HNSC	1	153251878	AGTTACCTT	-
		UCEC	2	153303342, 153253747	C, C	T, -
*FGFR1*	8	MEL	1	38282218	C	T
		CRC	2	38285864, 38285864	G, G	A, A
*KRAS*	12	CRC	1	25378706	C	A
*MLL2*	12	BLCA	3	49433217, 49433004, 49446856	CC, C, C	AA, A, T
		GBM	1	49416373	C	G
		HNSC	6	49446208, 49435775, 49428364, 49448535, 49428450, 49446699	C, T, C, C, C, T	A, C, T, T, G, C
		LUSC	7	49415826, 49443464, 49444670, 49436428, 49446494, 49442443, 49448310	C, C, T, C, T, T, C	G, A, A, -, A, C, T
		UCEC	1	49433506	C	T
*MLL3*	7	BLCA	3	151919766, 151891214, 151864230	A, C, C	G, G, T
		BRCA	5	152012424, 151904386, 151859199, 151859200, 152012425	C, T, -, A, T	A, C, A, -, G
		GBM	2	151962124, 151892993	T, T	C, C
		HNSC	2	151884562, 151891214	C, C	A, G
		KIRC	1	151848093	C	G
		LUAD	4	151871326, 151933018, 151850040, 151944986	A, C, C, C	C, A, G, A
		LUSC	1	151842380	G	T
		MEL	1	151871216	C	T
		UCEC	3	151866334, 152009030, 151896364	C, C, C	A, A, T
*MTOR*	1	GBM	1	11188183	C	T
		HNSC	1	11206848	C	A
		LUAD	2	11270872, 11292494	T, T	A, A
		MEL	1	11264758	G	A
*NF1*	17	BRCA	3	29508508, 29670148, 29588873	1-, TAAAAGG, TAGG	T, -, -
		DLBCL	1	29560018	A	G
		ESO	1	29554309	G	C
		GBM	7	29586049, 29685497, 29508439, 29684388, 29663349, 29508438, 29556484	G, G, G, GTAA, A, A, G	A, -, A, -, G, G, A
		HNSC	2	29548868, 29528502	G, A	T, T
		KIRC	1	29508512	G	T
		LUAD	11	29576138, 29562791, 29556992, 29585520, 29562627, 29548866, 29657518, 29557278, 29553702, 29665721, 29559717	G, G, G, G, A, A, T, G, G, G, G	A, T, T, C, T, T, A, T, T, C, T
		LUSC	2	29670025, 29663350	A, G	G, T
		MEL	1	29559899	GG	AA
		MM	1	29657517	G	A
		OV	1	29509525	G	T
		UCEC	3	29548949, 29654857, 29483145	T, G, G	C, A, A
*NOTCH1*	9	HNSC	9	139409741, 139413277, 139412204, 139397633, 139412204, 139407990, 139401756, 139392011, 139409935	C, C, C, C, C, C, C, C, -	G, A, T, T, A, T, T, T, TG,
		LUAD	1	139405257	C	A
		LUSC	2	139401756, 139401757	C, C	A, A
		UCEC	2	139401758, 139438554	C, C	A, A
*PBRM1*	3	HNSC	1	52595783	C	T
		KIRC	20	52685756, 52676059, 52661388, 52643328, 52676063, 52692333, 52678806, 52582081, 52702514, 52702660, 52696148, 52682360, 52662911, 52702662, 52661288, 52610715, 52663052, 52682459, 52677265, 52712615	A, T, T, C, T, T, C, A, C, T, C, CTT, T, C, C, C, C, C, G, T	G, -, A, T, A, A, T, -, T, A, A, -, -, T, A, G, A, T, -, C
		LUSC	2	52621527, 52623085	C, C	T, G
		UCEC	2	52643330, V	G, C	A, T
*PIK3CA*	3	BRCA	6	178917478, 178917478, 178928219, 178917478, 178942489, 178938775	G, G, -, G, C, T	A, A, ATA, A, T, A
		GBM	4	178916614, 178917478, 178952152, 178916614	A, G, A, A	G, A, G, G
		HNSC	1	178917478	G	A
		LUSC	1	178917478	G	A
		UCEC	8	178917478, 178916537, 178917478, 178917478, 178916614, 178917478, 178917478, 178917478	G, G, G, G, A, G, G, G	A, T, A, A, G, A, A, A
*PIK3R1*	5	GBM	5	67591246, 67591152, 67591246, 67591247, 67575431	A, T, A, GGT, -	G, -, G, -, A
		LUAD	1	67588928	G	T
		OV	1	67588086	G	C
		PRAD	1	67591246	A	G
		UCEC	9	67591154, 67591153, 67591246, 67588927, 67588927, 67591246, 67590504, 67591246, 67569842	T, G, TCAAAACTGTTTTTCAGGTGGTTGACTC, -, -, A, A, A, GTGA	1-, -, -, G, G, G, G, G, -
*PLAUR*	19	LUSC	1	44159726	C	T
*PTEN*	10	BRCA	5	89653784, 89711873, 89685316, 89712018, 89692976	A, A, T, T, TTCTATGGGGAAGTAAGGACCAGAGACAAAAAGGTAAG	T, G, -, C, -
		CRC	1	89711876	G	A
		GBM	9	89653779, 89725042, 89690846, 89685315, 89685314, 89720650, 89693009, 89720650, 89692768	1-, A, G, GTAA, T, G, G, G, A	AGAT, G, T, -, A, A, T, A, C
		HNSC	1	89712017	G	A
		KIRC	3	89712017, 89725043, 89725042	G, G, A	A, A, G
		LUAD	2	89720650, 89685269	G, G	A, T
		PRAD	1	89711874	G	A
		UCEC	21	89717609, 89685315, 89653780, 89685314, 89725043, 89717778, 89690801, 89725043, 89711875, 89725043, 89711875, 89720875, 89717609, 89712016, 89725042, 89720650, 89720876, 89711875, 89624305, 89690802, 89712017	G, GTAA, A, TG, G, T, A, G, G, G, G, G, G, AGTA, AATTTTCTTTCTCTAGGTGAAGCT, G, G, G, T, G, G	T, -, C, -, A, C, G, A, A, T, A, T, C, -, -, A, A, A, G, A, T
*RB1*	13	BLCA	4	48947629, 48947629, 49027248, 48947629	G, G, G, G	T, C, A, A
		CRC	1	49037867	A	T
		GBM	9	49039505, 49033823, 48953730, 49030485, 48953730, 48947629, 48916734, 48953730, 48951053	G, G, C, G, C, G, G, C, G	T, T, T, C, T, A, A, T, C
		HNSC	4	49033970, 48954379, 48955580, 48947540	G, T, G, G	T, G, A, A
		KIRC	1	49030485	G	A
		LUAD	7	49037972, 49027127, 48941739, 48916851, 48954377, 48939032, 48934152	G, A, G, G, A, -, G	A, G, T, T, T, A, -
		LUSC	5	48916733, 48916852, 48881542, 48916850, 48953728	A, T, G, G, A	G, G, T, T, G
		MEL	2	49030485, 48954300	G, G	A, A
		OV	1	48951053	G	C
		UCEC	2	49039506, 48947629	T, G	A, A
*SETD2*	3	BLCA	2	47142947, 47155366	C, G	T, A
		GBM	1	47147485	A	G
		KIRC	7	47079269, 47143045, 47155365, 47155365, 47059128, 47079155, 47161671	T, A, C, C, C, C, C	A, T, A, T, T, G, G
		LUAD	2	47129738, 47205413	C, A	T, G
		OV	1	47125871	A	T
		UCEC	1	47127805	C	A
*SMARCA4*	19	ESO	1	11113703	A	G
		KIRC	1	11129632	G	A
		LUAD	4	11141570, 11169565, 11107221, 11136097	G, G, G, G	T, T, T, T
		LUSC	1	11096082	G	T
*SPEN*	1	BLCA	2	16265921, 16247478	G, G	A, T
*SYK*	9	MEL	1	93607874	C	T
		LUSC	1	93636955	C	A
		KIRC	1	93624627	G	A
		UCEC	1	93641235	G	T
*TP53*	17	AML	4	7577609, 7579312, 7590694, 7578555	C, C, C, C	T, T, T, T
		BLCA	29	7573010, 7578555, 7578291, 7577610, 7577498, 7579311, 7577609, 7578370, 7579311, 7578175, 7578175, 7577610, 7578556, 7578555, 7577498, 7579312, 7577018, 7576852, 7578290, 7579312, 7578371, 7578553, 7578290, 7576851, 7576852, 7578554, 7579591, 7578556	T, C, T, T, C, C, C, C, C, -, A, T, T, C, C, C, C, C, C, C, C, T, T, C, A, C, A, C, T	C, G, A, C, T, A, T, A, T, CCTC, T, C, C, T, T, G, T, A, T, G, T, C, C, G, C, T, T, G, G
		CLL	2	7578370, 7572928	C, C	T, A
		CRC	6	7578290, 7576853, 7579313, 7579312, 7579590, 7579313	C, C, G, C, -, G	T, G, A, T, CT, A
		ESO	4	7579591, 7579311, 7579312, 7578554	C, C, C, A	T, T, T, C
		GBM	7	7576926, 7578555, 7577610, 7579312, 7579699, 7577609, 7578555	GC, C, T, C, C, C, C	AT, T, C, T, T, G, T
		HNSC	38	7578290, 7579311, 7577156, 7576927, 7576928, 7573010, 7578176, 7577017, 7577610, 7579310, 7579591, 7576853, 7578555, 7579312, 7578553, 7574034, 7578177, 7577498, 7576853, 7579698, 7576927, 7576852, 7573009, 7579698, 7577153, 7576852, 7578177, 7577018, 7579912, 7576853, 7576928, 7576840, 7577609, 7578553, 7579312, 7577153, 7578369, 7574034	C C, C, C, T, T, C, A, T, A, C, C, C, C, T, C, C, C, C, A, C, C, C, -, C, C, C, C, T, C, T, CAAGACTTAGTA, C, T, C, C, A, C	T, A, A, A, C, A, G, C, C, T, T, T, T, A, G, A, A, T, A, C, G, T, T, CC, A, T, T, A, C, T, C, -, T, C, A, A, C, A
		KIRC	1	7577498	C	A
		LUAD	24	7579699, 7578290, 7577156, 7578290, 7579312, 7577498, 7577609, 7577156, 7579312, 7578555, 7579312, 7577156, 7578175, 7578175, 7578556, 7576928, 7574034, 7579312, 7577153, 7578290, 7578370, 7577609, 7578177, 7577610	C, C, C, C, C, C, C, C, C, CT, C, C, A, A, T, T, C, C, C, C, C, C, C, T	A, A, A, G, A, T, A, T, A, AA, T, -, T, G, A, C, A, A, A, A, A, G, A, C
		LUSC	18	7579312, 7577609, 7579312, 7578177, 7578556, 7578177, 7579311, 7576928, 7579312, 7579312, 7578176, 7576853, 7576851, 7577156, 7574035, 7579311, 7578177, 7578370	C, C, C, C, T, C, C, T, C, C, C, C, A, C, T, C, C, C	A, A, A, G, C, T, A, A, A, A, A, G, T, A, C, A, T, A
		MEL	2	7576855, 7578555	G, C	A, A
		MM	1	7578555	C	G
		OV	36	7577609, 7578555, 7578556, 7578290, 7578555, 7576852, 7578266, 7578290, 7576852, 7579312, 7578555, 7577498, 7578369, 7579311, 7577498, 7578370, 7574034, 7576852, 7578290, 7577018, 7576852, 7576927, 7578556, 7579592, 7578370, 7576927, 7578370, 7577610, 7578555, 7579311, 7578177, 7578176, 7578176, 7578176, 7576852, 7577019	C, C, T, C, C, C, TAAGATGCTGAGGAGGGGCCAGACC, C, C, C, C, C, A, C, C, C, C, C, C, C, C, C, T, T, C, C, C, T, C, C, C, C, C, C, C, CT	T, T, C, T, A, T, -, G, A, A, A, T, C, A, A, A, T, T, T, T, G, T, C, A, A, T, T, C, T, A, T, A, T, A, A, -
		UCEC	1	7574034	C	G
*VHL*	3	KIRC	27	10188321, 10183872, 10183872, 10183873, 10188197, 10188321, 10191470, 10188197, 10191470, 10191469, 10191470, 10188197, 10191470, 10191469, 10188196, 10191469, 10188321, 10183871, 10183871, 10183871, 10188321, 10183872, 10188190, 10188320, 10191648, 10188322, 10188319	G, G, G, T, G, G, G, G, G, A, G, G, G, A, A, A, G, G, G, G, G, G, CCCGATA, GGTAC, G, T, AGGTACTGACGTTTTACTTTTTAAAA	C, A, C, C, T, T, A, C, A, G, C, T, T, G, T, G, T, C, -, C, T, A, -, -, T, C, -
		MEL	1	10188320	G	A

Information retrieved from the Tumor Portal (http://www.tumorportal.org). * AML, Acute myeloid leukemia; BLCA, Bladder; BRCA, Breast; CARC, Carcinoid; CLL, Chronic lymphocytic leukemia; CRC, Colorectal; DLBCL, Diffuse large B-cell lymphoma; ESO, Esophageal adenocarcinoma; GBM, Glioblastoma multiforme; HNSC, Head and neck; KIRC, Kidney clear cell; LUAD, Lung adenocarcinoma; LUSC, Lung squamous cell carcinoma; MED, Medulloblastoma; MEL, Melanoma; MM, Multiple mieloma; NB, Neuroblastoma; OV, Ovarian; PanCan, combined cohort; PRAD, Prostate; RHAB, Rhabdoid tumor; UCEC, Endometrial.

**Table 3:** Current patents in alternative splicing and cancer.

Patent ID	Institution	Cancer	Observation	Patent	Reference
US20170108504A1	Mumetel	Colon cancer cell lines (LS174T, LoVo adn HCT116 cell lines).	They describe a new Bax isoform, BaxΔ2. The BaxΔ2 isoform resulted from combination of Bax microsatellite mutation and alternative splicing Bax exon 2. It is also discovered that BaxΔ2 only exists in the Bax mutated cells and renders cancer cells sensitive to certain chemotherapeutic drugs that target caspase 8.	The patent claims a method for detection the expression of the BaxΔ2 protein in a cancer cell isolated from the patient. The detection can use an antibody having specificity to the BaxΔ2 protein, or alternatively by detecting a RNA sequence encoding the BaxΔ2 protein.	[153]
US2016333426A1	Chiba University	colon cancer cell line (LS180), human colorectal cancer cell line (HCT116) and human pancreatic cancer cell line (PK45p) [2].	OATP1B3 is a transporter expressed on the cell membrane that is involved in uptake of various compounds comprising anti-cancer drugs into a cell. Has been reported that the expression and the function of OATP1B3 affect patient's survival rate in breast cancer and prostate cancer.	Describes a method for measuring an alternative splicing variant of organic anion transporting polypeptide 1B3 (OATP1B3) mRNA in a sample of cancer patient.	[154]
US20160208337A1	International Medical Researchg Cancer	breast tissue samples.	Lamin A mRNA ratio is increased in breast cancer and this mRNA ratio may be of diagnostic use in all clinical stages of breast cancer.	Describes a method for detecting cancer by determining ratios of alternatively spliced Lamin A/C gene mRNAs especially an increased.	[155]
US2014364483A1	George Washington University	Prostate cancer.	Study of alternative splicing variants for genes in the oncogenic signaling pathways, such as *PIK3CD*, *FGFR3*, *TSC2, FGFR2*, *PDGFRA*, *ITGA4*, *MET*, *EPHA3*, *NF1*, *RASGRP2*, *CTNNB1*, *TSC2* , *ATM*, *CDK4*, and *RB1*. These novel splicing variants are particularly useful for the detection due to the importance of these genes in oncogenic signaling pathways.	Describes a method for quantitative analysis of the expression profiles of *PIK3CD*, *FGFR3*, *TSC2*, *RASGRP2*, *ITGA4*, *MET*, *NF1* and *BAK1* in prostate samples confirm differential splicing between the African Americans (AA) and Caucasian American (CA) patients.	[156]
US20060292577	Purdue Research Foundation	MCF-10A (mammary non-cancer), BT-20 (mammary cancer), and HeLa (cervical cancer) cells.	Describes a cancer-specific alternatively spliced tumor-specific plasma membrane NADH oxidase/thiol interchange protein transcript termed E4mtNOX herein.	Method for silencing exon 4 in E4mtNOX2	[157]

**Table 4:** Current clinical trials (phase III) for cancer patients that involve antisense therapy.

Study Title	Conditions	Interventions	Locations
Daunorubicin Hydrochloride, Cytarabine and Oblimersen Sodium in Treating Patients with Previously Untreated Acute Myeloid Leukemia	Adult Acute Myeloid Leukemia With 11q23 (MLL) Abnormalities Adult Acute Myeloid Leukemia With Inv (16) (p13;q22) Adult Acute Myeloid Leukemia With t (15;17) (q22;q12)	Biological: oblimersen sodium Drug: cytarabine Drug: daunorubicin hydrochloride Other: laboratory biomarker analysis	Cancer and Leukemia Group B Chicago, Illinois, United States Arthur G. James Cancer Hospital and Solove Research Institute at Ohio State University Medical Center Columbus, Ohio, United States
Dacarbazine With or Without Oblimersen (G3139) in Treating Patients with Advanced Malignant Melanoma	Melanoma (Skin)	Biological: oblimersen sodium Drug: dacarbazine	Jonsson Comprehensive Cancer Center, UCLA Los Angeles, California, United States Genta Incorporated Berkeley Heights, New Jersey, United States
Dexamethasone with or without Oblimersen in Treating Patients with Relapsed or Refractory Multiple Myeloma	Multiple Myeloma and Plasma Cell Neoplasm	Biological: oblimersen sodium Drug: dexamethasone	Genta Incorporated Berkeley Heights, New Jersey, United States
Fludarabine and Cyclophosphamide with or without Oblimersen in Treating Patients with Relapsed or Refractory Chronic Lymphocytic Leukemia	Leukemia	Biological: filgrastim Biological: oblimersen sodium Drug: cyclophosphamide Drug: fludarabine phosphate	Genta Incorporated Berkeley Heights, New Jersey, United States
Carboplatin and Paclitaxel with or Without ISIS 3521 in Treating Patients with Non-Small Cell Lung Cancer	Lung Cancer	Biological: ISIS 3521 Drug: carboplatin Drug: paclitaxel	ISIS Pharmaceuticals, Inc. Carlsbad, California, United States Ireland Cancer Center Cleveland, Ohio, United States
Pharmacokinetics of G3139 in Subjects with Advanced Melanoma, Including Those with Normal Hepatic Function and Those with Moderate Hepatic Impairment	Advanced Melanoma and Normal or Impaired Hepatic Function	Drug: Genasense^®^ (G3139, oblimersen sodium)	
Docetaxel with or Without Oblimersen in Treating Patients with Non-Small Cell Lung Cancer	Lung Cancer	Biological: oblimersen sodium Drug: docetaxel	University of Alabama at Birmingham Comprehensive Cancer Center Birmingham, Alabama, United States Montgomery Cancer Center Montgomery, Alabama, United States Little Rock Hematology-Oncology Associates Little Rock, Arkansas, United States
A Study Evaluating the Pain Palliation Benefit of Adding Custirsen to Docetaxel Retreatment or Cabazitaxel as Second Line Therapy in Men with Metastatic Castrate Resistant Prostate Cancer (mCRPC)	Castrate-Resistant Prostate Cancer Hormone Refractory Prostate Cancer	Drug: custirsen sodium Drug: isotonic, 0.9% sodium chloride Drug: docetaxel	La Verne, California, United States Ft. Lauderdale, Florida, United States Tampa, Florida, United States
Trial of Dacarbazine with or without Genasense in Advanced Melanoma	Melanoma	Drug: dacarbazine plus Genasense Drug: dacarbazine plus placebo	University of South Alabama Hospital, Mitchell Cancer Institute Mobile, Alabama, United States San Diego Pacific Oncology and Hematology Associates Inc. Encinitas, California, United States Redwood Regional Medical Group, Inc. Santa Rosa, California, United States
Efficacy and Safety of AP 12009 in Patients with Recurrent or Refractory Anaplastic Astrocytoma or Secondary Glioblastoma	Anaplastic Astrocytoma Glioblastoma	Drug: trabedersen Drug: temozolomide Device: Drug delivery system for administration of AP 12009	NJ Neuroscience Institute; JFK Medical Center Edison, New Jersey, United States Winthrop University Hospital Mineola, New York, United States University of Rochester Medical Center Rochester, New York, United States

Information retrieved from ClinicalTrials.gov.
